# The Recognition Pathway of the SARS-CoV-2 Spike Receptor-Binding Domain to Human Angiotensin-Converting Enzyme 2

**DOI:** 10.3390/molecules29081875

**Published:** 2024-04-19

**Authors:** Can Peng, Xinyue Lv, Zhiqiang Zhang, Jianping Lin, Dongmei Li

**Affiliations:** 1 State Key Laboratory of Medicinal Chemical Biology, College of Pharmacy and Tianjin Key Laboratory of Molecular Drug Research, Nankai University, Haihe Education Park, 38 Tongyan Road, Tianjin 300350, China; 2120211306@mail.nankai.edu.cn (C.P.); 2120211384@mail.nankai.edu.cn (X.L.); 2 Xiongan Institute of Innovation, Xiong’an New Area 070001, China; zhangzhiqiang@xii.ac.cn

**Keywords:** Su-GaMD simulations, SARS-CoV-2, ACE2, protein–protein recognition pathway, enhanced sampling

## Abstract

COVID-19 caused by SARS-CoV-2 has spread around the world. The receptor-binding domain (RBD) of the spike protein of SARS-CoV-2 is a critical component that directly interacts with host ACE2. Here, we simulate the ACE2 recognition processes of RBD of the WT, Delta, and OmicronBA.2 variants using our recently developed supervised Gaussian accelerated molecular dynamics (Su-GaMD) approach. We show that RBD recognizes ACE2 through three contact regions (regions I, II, and III), which aligns well with the anchor–locker mechanism. The higher binding free energy in State d of the RBD_OmicronBA_._2_-ACE2 system correlates well with the increased infectivity of OmicronBA.2 in comparison with other variants. For RBD_Delta_, the T478K mutation affects the first step of recognition, while the L452R mutation, through its nearby Y449, affects the RBD_Delta_-ACE2 binding in the last step of recognition. For RBD_OmicronBA_._2_, the E484A mutation affects the first step of recognition, the Q493R, N501Y, and Y505H mutations affect the binding free energy in the last step of recognition, mutations in the contact regions affect the recognition directly, and other mutations indirectly affect recognition through dynamic correlations with the contact regions. These results provide theoretical insights for RBD-ACE2 recognition and may facilitate drug design against SARS-CoV-2.

## 1. Introduction

The emergence of COVID-19 triggered a global pandemic. COVID-19 is caused by the severe acute respiratory syndrome coronavirus 2 (SARS-CoV-2) infection of the human body. By December 2023, COVID-19 had caused nearly 7 million deaths [1] and seriously affected the international economy. The wild type (WT) SARS-CoV-2 was first identified in late December 2019 [2]; it spread rapidly worldwide, and gave rise to the pandemic. For several years following the pandemic, many variants of concern (VOCs) of SARS-CoV-2 were declared by the World Health Organization (WHO). The Delta variant (B.1.617.2) identified in October 2020 showed increased transmissibility and disease severity compared to the WT [3]. Omicron (B.1.1.529) variants, including BA.1, BA.2, BA.3, BA.4, and BA.5, were first observed in November 2021; they were more infectious than all of the previous variants and became the dominant VOC around the world [4,5,6,7,8].

SARS-CoV-2 is a positive-sense single-stranded RNA virus [9], and the SARS-CoV-2 particle is composed of four auxiliary and structural proteins, including a spike protein, envelope protein, membrane protein, and nucleocapsid protein [10]. Angiotensin-converting enzyme II (ACE2) is an entry receptor [11] on the host cell surface, which provides the entry point for SARS-CoV-2 to hook into and infect the host cells. SARS-CoV-2 mainly uses its spike proteins to recognize ACE2 and mediate SARS-CoV-2 to enter into the human body [12]. The receptor-binding domain (RBD) of the spike protein is a critical functional component that directly interacts with ACE2 on the host cell membrane [13]. So far, it is well-established that host susceptibility to SARS-CoV-2 is mainly determined by the binding of the viral spike RBD to ACE2 during the initial viral attachment step [14,15]. Therapeutic agents that disrupt the binding of spike RBD and ACE2 would slow, or even block, SARS-CoV-2’s infection of the host cells [14,16,17] and counteract its infectivity. Therefore, it is very important to understand the details of the recognition and binding between spike RBD and ACE2.

The RBD of the Delta variant (RBD_Delta_) possesses L452R and T478K mutations, while the RBD of the Omicron variants (RBD_Omicron_) has accumulated at least 15 mutations [18,19]. Due to the role of the RBD in ACE2 recognition and binding, it stands to reason that mutations in RBD can dramatically impact spike binding for ACE2 and, ultimately, SARS-CoV-2 infectivity [20]. So far, many crystal structures and cryo-EM structures of RBD-ACE2 complexes have been reported, including the WT and Delta and Omicron variants [14,18,21,22]. From these crystal structures, we can learn some details of the intermolecular interaction between RBD and ACE2, as well as some changes caused by residue mutations. However, RBD-ACE2 recognition and binding is a dynamic process. Molecular dynamics (MD) simulation is usually a supplement to traditional structural research used to observe the dynamics of the protein–protein recognition processes at the atomic level [23]. Many studies investigated the interaction interface between RBD and ACE2 through MD simulations [24,25,26,27,28,29]. For example, Kodchakorn et al. [24] performed conventional MD (cMD) simulations on the RBD-ACE2 complexes for the WT, Kappa, Delta, and Omicron variants of SARS-CoV-2 and identified the hotspot residues at the RBD-ACE2 interface. cMD simulations carried out by Pitsillou et al. [25] indicated that RBD_Delta_ and RBD_Omicron_ bind to ACE2 with similar affinities and are stronger than RBD_WT_. However, these studies only characterized the binding interfaces and binding affinities between RBD and ACE2. The dynamic binding process from free RBD and ACE2 in the solvent to the RBD-ACE2 complex was not simulated by these cMD studies due to the long timescale of the binding process and the computation expensiveness. In 2021, Cong et al. [26] proposed the anchor–locker recognition mechanism involved in the binding of the spike RBD to ACE2 and validated the dissociation process of RBD and ACE2 through umbrella sampling simulations. Following this, Kim et al. [27] investigated the interactions between Epsilon, Kappa, Alpha, Beta, Gamma, Delta, and Omicron spike RBD and ACE2 using steered molecular dynamic (SMD) simulations. But these studies only tracked the unbinding process of spike RBD and ACE2 by using enhanced sampling simulations (e.g., umbrella sampling and SMD) with additional constraints.

To investigate the binding process of spike RBD to ACE2, Chen et al. [30] performed metadynamics simulations on RBD and ACE2, and they characterized the free energy landscape and elucidated the binding mechanism of spike RBD to ACE2 with and without heparan sulfate fragment DP4. In 2021, Deganutti et al. [31] simulated the binding process of spike RBD_WT_ to ACE2 using the supervised MD (SuMD) approach. In addition, they explored the molecular recognition of different variants of RBD to ACE2 through SuMD simulations and elucidated the impact of mutations [32].

In the present study, by using our recently developed supervised Gaussian accelerated molecular dynamics (Su-GaMD) method [33], which incorporates a tabu-like supervision algorithm into a Gaussian accelerated molecular dynamics (GaMD) [34] simulation, we simulated the binding process of spike RBD to ACE2. The possible recognition pathways were revealed, important intermediate states were identified, and the RBD_WT_-ACE2, RBD_Delta_-ACE2 and RBD_OmicronBA_._2_-ACE2 complexes were reconstructed at the end of the simulations. The recognition mechanisms of RBD to ACE2 for the WT, Delta, and OmicronBA.2 variants of SARS-CoV-2 were revealed, and the effects of the mutations in RBD_Delta_ and RBD_OmicronBA_._2_ to the RBD-ACE2 recognition and binding were discussed.

## 2. Results and Discussion

### 2.1. Recognition Process of Spike RBD of Different Variants to ACE2

**Figure 1:**
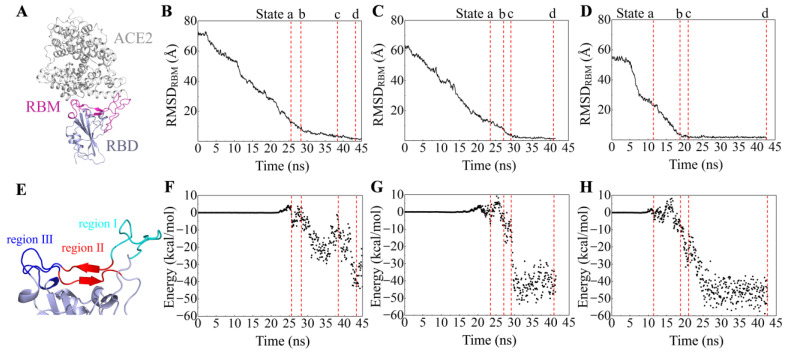
(**A**) Representation of the receptor-binding motif (RBM) in the RBD-ACE2 complex. The RBM is colored in magenta. Time-dependent RMSD_RBM_ in the recognition process of (**B**) RBD_WT_, (**C**) RBD_Delta_, and (**D**) RBD_OmicronBA_._2_ to ACE2. (**E**) The designation of region I (colored in cyan), II (colored in red), and III (colored in blue) for the spike RBD. Time-dependent binding free energies for (**F**) RBD_WT_, (**G**) RBD_Delta_, and (**H**) RBD_OmicronBA_._2_ to ACE2 during the recognition process. The red dashed lines represent States a, b, c and d.

To investigate the binding process of spike RBDs of different variants to ACE2, we performed Su-GaMD simulations on three systems (i.e., RBD_WT_-ACE2, RBD_Delta_-ACE2, and RBD_OmicronBA_._2_-ACE2) by placing RBD > 40 Å away from ACE2. Starting from the completely dissociated RBD and ACE2, we reconstructed the RBD_WT_-ACE2, RBD_Delta_-ACE2, and RBD_OmicronBA_._2_-ACE2 complexes by supervising the RMSD of the heavy atoms in the main chain of the receptor-binding motif (RBM, residues 438–506 in RBD, as shown in Figure 1A) relative to the targeting structures (RMSD_RBM_) in the Su-GaMD simulations. The RMSD_RBM_ in the simulations of the RBD-ACE2 recognition process are shown in Figure 1B–D. The binding free energies between RBD and ACE2 were calculated during the recognition process (Figure 1F–H).

**Figure 2:**
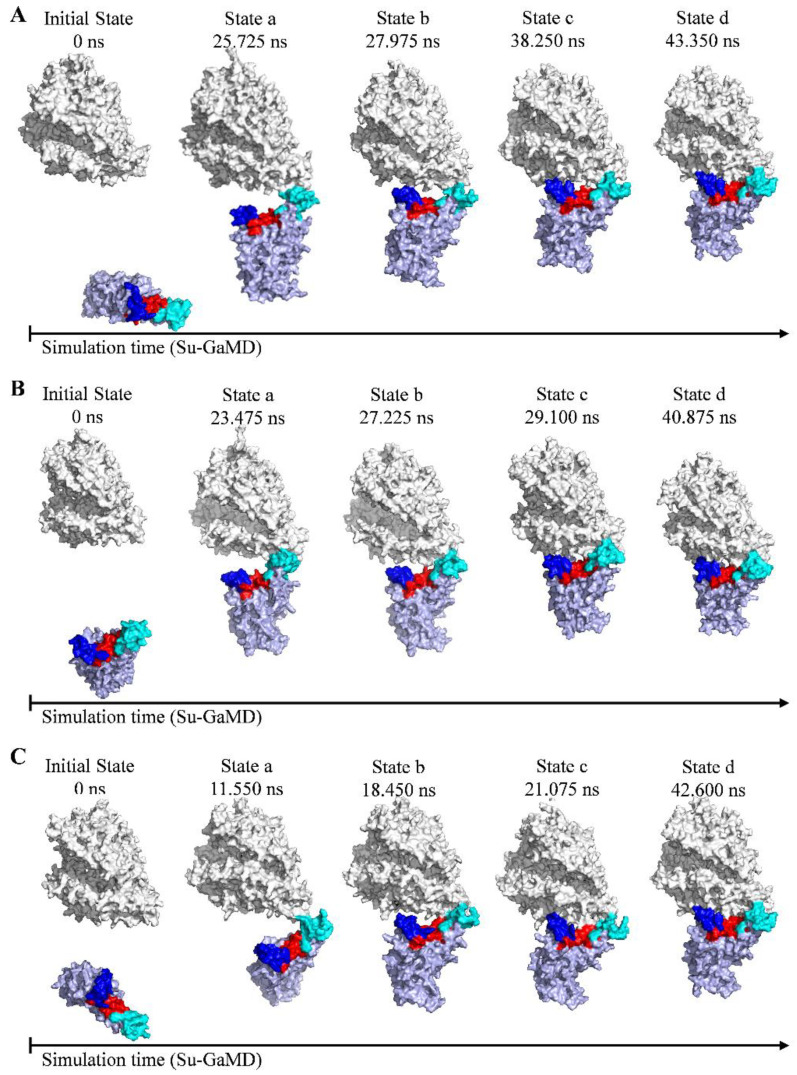
Schematic representations of the recognition process of (**A**) RBD_WT_, (**B**) RBD_Delta_, and (**C**) RBD_OmicronBA_._2_ to ACE2. ACE2 and RBD are colored in gray and light blue, respectively. Regions I, II, and III of RBD are colored in cyan, red, and blue.

To depict the recognition process of RBD to ACE2, the contact motif of RBD to ACE2 is divided into three regions. Region I is a loop composed of residues 473 to 490, region II is composed of two β-sheets (residues 450 to 456 and residues 491 to 495), and region III is another loop composed of residues 444 to 449 and residues 496 to 505 (Figure 1E). We selected four presentative states (States a, b, c, and d) along the simulation time to depict the RBD-ACE2 recognition pathway for each of the SARS-CoV-2 variants. Schematic representations of the recognition process of RBD_WT_, RBD_Delta_, and RBD_OmicronBA_._2_ to ACE2 are shown in Figure 2.

For the RBD_WT_-ACE2 system, the RMSD_RBM_ gradually decreases from 72.7 Å to 1.9 Å (Figure 1B) in the Su-GaMD simulation. Through the Su-GaMD simulation, RBD_WT_ gradually comes close to ACE2 and the RBD_WT_-ACE2 complex is constructed at the end of the simulation. The dynamic recognition of RBD_WT_ to ACE2 is observed on the basis of the Su-GaMD trajectory (Video S1).

As shown in Figure 2A, in State a, RBD_WT_ contacts ACE2 through region I. In State b, RBD_WT_ contacts ACE2 regions I and III. In State c, RBD_WT_ contacts ACE2 through regions I, II, and III. In State d, the RBD_WT_-ACE2 complex is constructed. This RBD_WT_-ACE2 complex aligns well with the crystal structure 6M0J (with an RMSD of 2.0 Å for RBD_WT_, an RMSD of 2.2 Å for ACE2, and an RMSD of 1.9 Å for the whole RBD_WT_-ACE2 complex). To sum up, in the recognition process, RBD_WT_ is anchored to ACE2 through region I in the first step (State a), and then region III at the other end of RBD_WT_ is locked to ACE2 as well (State b); finally, region II in the middle of RBD_WT_ is attached to ACE2 and reinforces the binding (State c), so that the entire RBD_WT_ is closely bound to ACE2 (State d) and the RBD_WT_-ACE2 complex is constructed (Figure 2A). This recognition process revealed by our Su-GaMD simulation corresponds well with the anchor–locker mechanism [26] proposed by Cong et al. This anchor–locker mechanism was supported by an umbrella sampling simulation of the RBD-ACE2 dissociation process in their work, and the present Su-GaMD simulation confirms the rationality of this mechanism through the Su-GaMD simulation of the RBD-ACE2 recognition process.

To evaluate the statistic of the simulations, the Su-GaMD production runs were conducted from three different starting points of RBD_WT_ to ACE2. The initial state, State a, and the RMSD_RBM_ of the three independent Su-GaMD simulations of the RBD_WT_-ACE2 recognition process are shown in Figure S1. It is seen that RBD_WT_ can be anchored to ACE2 through region I (see State a) and achieve a final RBD_WT_-ACE2 complex similar to the crystal structure 6M0J (with RMSD_RBM_ of 1.9, 1.8, and 1.2 Å) in the Su-GaMD simulations from three different starting points.

Moreover, for comparison, we performed a 1000-ns cMD simulation from the same starting point as in Figure 2A. It is seen that the RBD_WT_-ACE2 complex similar to the crystal structure 6M0J cannot be reached in this extremely long-time cMD simulation (the final RMSD_RBM_ is around 50 Å, see Figure S2). Thus, we can reconstruct the RBD_WT_-ACE2 complex in a binding mode similar to that of the crystal structure 6M0J and observe the RBD_WT_-ACE2 recognition process at the nanosecond timescale by using the Su-GaMD strategy, while this RBD_WT_-ACE2 complex cannot be reached even during a long-time (e.g., 1000 ns) cMD simulation.

**Figure 3:**
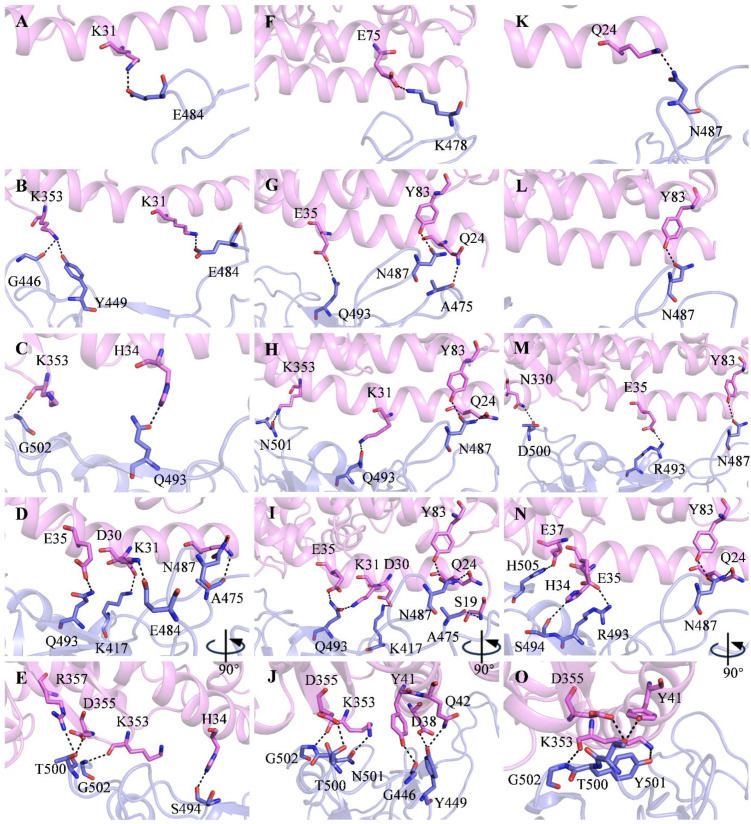
(**A**–**E**) Detailed residue interactions between RBD and ACE2 in States a to d for the RBD_WT_-ACE2 system. (**F**–**J**) Detailed residue interactions between RBD and ACE2 in States a to d for the RBD_Delta_-ACE2 system. (**K**–**O**) Detailed residue interactions between RBD and ACE2 in States a to d for the RBD_OmicronBA_._2_-ACE2 system. ACE2 and RBD are colored in violet and slate, residues in the ACE2-RBD interface are shown as sticks. Hydrogen bonds and salt bridges between RBD and ACE2 are shown as dashed lines. The donor–acceptor heavy atom distance range of hydrogen bonds is 2.5 to 3.5 Å, and the salt bridges are interactions of amino acids with opposing charge where at least two heavy atoms lie within 3.5 Å.

The detailed residue interactions between RBD and ACE2 through the recognition pathway are depicted in Figure 3. In State a, RBD_WT_ recognizes ACE2 with a salt bridge between E484^RBD^ and K31^ACE2^ (Figure 3A). Thus, E484^RBD^ is the key residue in the first step of the RBD_WT_ recognition of ACE2. In State b, besides the E484^RBD^-K31^ACE2^ salt bridge, RBD_WT_ interacts with ACE2 through two hydrogen bonds, G446^RBD^-K353^ACE2^ and Y449^RBD^-K353^ACE2^ (Figure 3B). In State c, RBD_WT_ binds ACE2 through hydrogen bonds Q493^RBD^-H34^ACE2^ and G502^RBD^-K353^ACE2^ (Figure 3C). In State d, RBD_WT_ interacts with ACE2 through salt bridges K417^RBD^-D30^ACE2^ and E484^RBD^-K31^ACE2^ and hydrogen bonds A475^RBD^-Q24^ACE2^, N487^RBD^-Q24^ACE2^, Q493^RBD^-E35^ACE2^, S494^RBD^-H34^ACE2^, T500^RBD^-D355^ACE2^, T500^RBD^-R357^ACE2^, and G502^RBD^-K353^ACE2^ (Figure 3D,E).

For the RBD_Delta_-ACE2 system (Video S2), the RMSD_RBM_ gradually decreases from 63.5 Å to 1.7 Å (Figure 1C) in the Su-GaMD simulation. In the recognition process (Figure 2B), RBD_Delta_ is anchored to ACE2 through region I in the first step (State a). Then, besides region I, region II in the middle of RBD_Delta_ is attached to ACE2 (State b). After that, region III at the other end of RBD_Delta_ is locked to ACE2 as well and reinforces the binding (State c) so that the entire RBD_Delta_ is closely bound to ACE2 through regions I, II, and III (State d). This recognition process is slightly different from that of RBD_WT_. RBD_WT_ recognizes ACE2 in the chronological order of regions I, III, and II, while RBD_Delta_ recognizes ACE2 in the chronological order of regions I, II, and III. Su-GaMD production runs conducted from three different starting points of RBD_Delta_ to ACE2 show that RBD_Delta_ can be anchored to ACE2 through region I (see State a) and achieve a final RBD_Delta_-ACE2 complex similar to the cryo-EM structure 7W9I (with RMSD_RBM_ of 1.7, 1.8, and 1.3 Å) (Figure S3). The constructed RBD_Delta_-ACE2 complex in the Su-GaMD simulation aligns well with the cryo-EM structure 7W9I (with an RMSD of 2.0 Å for RBD_Delta_, an RMSD of 2.6 Å for ACE2, and an RMSD of 2.1 Å for the whole RBD_Delta_-ACE2 complex). In State a, RBD_Delta_ recognizes ACE2 with a salt bridge between K478^RBD^ and E75^ACE2^ (Figure 3F). Thus, K478^RBD^ is the key residue in the first step of the RBD_Delta_ recognition of the ACE2. In State b, the K478^RBD^-E75^ACE2^ salt bridge formed in State a is broken, and RBD_Delta_ interacts with ACE2 through hydrogen bonds A475^RBD^-Q24^ACE2^, N487^RBD^-Y83^ACE2^, and Q493^RBD^-E35^ACE2^ (Figure 3G). In State c, RBD_Delta_ binds with ACE2 through hydrogen bonds N487^RBD^-Y83^ACE2^, N487^RBD^-Q24^ACE2^, Q493^RBD^-K31^ACE2^, and N501^RBD^-K353^ACE2^ (Figure 3H). In State d, RBD_Delta_ interacts with ACE2 through salt bridge K417^RBD^-D30^ACE2^ and hydrogen bonds G446^RBD^-Y41^ACE2^, Y449^RBD^-D38^ACE2^, Y449^RBD^-Q42^ACE2^, A475^RBD^-S19^ACE2^, N487^RBD^-Q24^ACE2^, N487^RBD^-Y83^ACE2^, Q493^RBD^-K31^ACE2^, Q493^RBD^-E35^ACE2^, T500^RBD^-D355^ACE2^, N501^RBD^-K353^ACE2^, and G502^RBD^-K353^ACE2^ (Figure 3I,J).

For the RBD_OmicronBA_._2_-ACE2 system (Video S3), the RMSD_RBM_ gradually decreases from 55.4 Å to 1.2 Å (Figure 1D) in the Su-GaMD simulation. In the recognition process (Figure 2C), RBD_OmicronBA_._2_ is anchored to ACE2 through region I in the first step (State a). Then, besides region I, region II in the middle of RBD_Delta_ is attached to ACE2 (State b). After that, in addition to regions I and II, region III at the other end of RBD_Delta_ is locked to ACE2 and reinforces the binding (State c), so that the entire RBD_OmicronBA_._2_ is closely bound to ACE2 through regions I, II, and III (State d). This recognition process, in the chronological order of regions I, II, and III of RBD_OmicronBA_._2_, is the same as that of RBD_Delta_ and is slightly different from that of RBD_WT_. Su-GaMD production runs conducted from three different starting points of RBD_OmicronBA_._2_ to ACE2 show that RBD_OmicronBA_._2_ can be anchored to ACE2 through region I (see State a) and achieve a final RBD_OmicronBA_._2_-ACE2 complex similar to the crystal structure 7ZF7 (with RMSD_RBM_ of 1.2, 1.4 and 1.8 Å) (Figure S4). The constructed RBD_OmicronBA_._2_-ACE2 complex in the Su-GaMD simulation aligns well with the crystal structure 7ZF7 (with an RMSD of 1.6 Å for RBD_OmicronBA_._2_, an RMSD of 2.5 Å for ACE2 and an RMSD of 2.1 Å for the whole RBD_OmicronBA_._2_-ACE2 complex). In State a, RBD_OmicronBA_._2_ recognizes ACE2 with a hydrogen bond between N487^RBD^ and Q24^ACE2^ (Figure 3K). Thus, N487^RBD^ is the key residue in the first step of the RBD_OmicronBA_._2_ recognition of ACE2. In State b, RBD_OmicronBA_._2_ interacts with ACE2 through hydrogen bond N487^RBD^-Y83^ACE2^ (Figure 3L). In State c, RBD_OmicronBA_._2_ binds with ACE2 through salt bridge R493^RBD^-E35^ACE2^ and hydrogen bonds N487^RBD^-Y83^ACE2^ and D500^RBD^-N330^ACE2^ (Figure 3M). In State d, RBD_OmicronBA_._2_ interacts with ACE2 through salt bridge R493^RBD^-E35^ACE2^ and hydrogen bonds N487^RBD^-Q24^ACE2^, N487^RBD^-Y83^ACE2^, S494^RBD^-H34^ACE2^, T500^RBD^-Y41^ACE2^, T500^RBD^-D355^ACE2^, Y501^RBD^-K353^ACE2^, G502^RBD^-K353^ACE2^, and H505^RBD^-E37^ACE2^ (Figure 3N,O).

In State d, the binding free energies of RBD to ACE2 in the systems RBD_WT_-ACE2, RBD_Delta_-ACE2, and RBD_OmicronBA_._2_-ACE2 are −42.7, −43.1, and −48.8 kcal/mol, respectively (State d in Figure 1F–H). These binding free energies suggest that the binding affinity between RBD_Delta_ and ACE2 is a little stronger than RBD_WT_, while the binding affinity between RBD_OmicronBA_._2_ and ACE2 is much stronger than both RBD_WT_ and RBD_Delta_. This aligns well with the enhanced infectivity of the OmicronBA.2 variant compared to all of the previous variants.

### 2.2. Effect of the Mutations on Spike RBD to the RBD_Delta_-ACE2 Recognition

It is known that RBD_Delta_ possesses the L452R and T478K mutations. To investigate the effect of the mutations on the RBD_Delta_-ACE2 recognition process, we analyzed the residue interactions in the first step (State a) and the residue contributions to the binding free energies of the constructed RBD_Delta_-ACE2 complex (State d) in detail.

In the first step of recognition, RBD_WT_ recognizes ACE2 with a salt bridge through E484^RBD^ (Figure 3A). Due to RBD_Delta_’s T478K mutation (which is longer), the first step in recognizing ACE2 for RBD_Delta_ is the salt bridge, not through E484^RBD^, but K478^RBD^ (Figure 3F).

**Figure 4:**
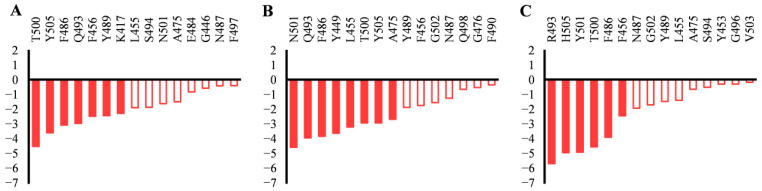
Binding free energy decomposition of residues in the binding interface of (**A**) RBD_WT_, (**B**) RBD_Delta_, and (**C**) RBD_OmicronBA_._2_ in State d.

**Figure 5:**
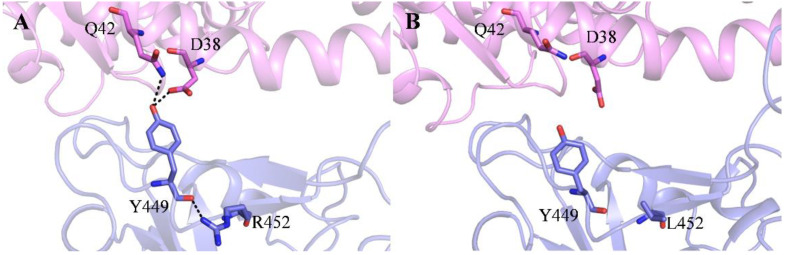
(**A**) Effect of the L452R mutation in orienting Y449 and strengthening the binding between RBD_Delta_ and ACE2, and (**B**) the corresponding residues in RBD_WT_ and ACE2. Hydrogen bonds are shown as dashed lines. The donor–acceptor heavy atom distance range of hydrogen bonds is 2.5 to 3.5 Å.

To provide more detailed and microlevel information about the binding between RBD and ACE2 in State d, the binding free energies were decomposed to residues. The residues in the binding interface of RBD that contribute the most to the binding free energies in State d are listed in Figure 4. In State d, the residues that contribute the most (<−2 kcal/mol) to the binding free energy of RBD_WT_ are T500, Y505, F486, Q493, K456, Y489, and K417 (Figure 4A), while the residues that contribute the most to the binding free energy of RBD_Delta_ are N501, Q493, F486, Y449, L455, T500, Y505, and A475 (Figure 4B). We can see that the L452R mutation in RBD_Delta_ does not contribute directly to the binding free energy in State d. But when we look closely at the interaction of RBD_Delta_ and ACE2 in State d (Figure 5A), we find that the R452 forms a hydrogen bond with its nearby Y449, which makes Y449 possess a proper orientation and form hydrogen bonds with D38 and Q42 of ACE2, thus strengthening the binding between RBD_Delta_ and ACE2.

To sum up, the T478K mutation affects the first step in the RBD_Delta_ recognition of ACE2, while the L452R mutation affects, not directly but through its nearby Y449, the binding between RBD_Delta_ and ACE2 in the last step of recognition.

### 2.3. Effect of the Mutations on Spike RBD to RBD_OmicronBA_._2_-ACE2 Recognition

RBD_OmicronBA_._2_ accumulates the G339D, S371F, S373P, S375F, T376A, D405N, R408S, K417N, N440K, S477N, T478K, E484A, Q493R, Q498R, N501Y, and Y505H mutations. To investigate the effect of the mutations on the RBD_OmicronBA_._2_-ACE2 recognition process, we analyzed the residue interactions in the first step (State a), the residue contributions to the binding free energies of the constructed RBD_OmicronBA_._2_-ACE2 complexes (State d), and the dynamic cross-correlations in RBD.

In RBD_OmicronBA_._2_, E484 mutates to A484 (shorter and uncharged). In the first recognition step, RBD_OmicronBA_._2_ is different from RBD_WT_, it recognizes ACE2 not through the E484^RBD^ salt bridge, but through another residue, N487^RBD^ (Figure 3K). The E484A mutation not only affects the first step of the recognition of RBD_OmicronBA_._2_ to ACE2, but also plays a crucial role in antibody recognition. The E484A mutation in RBD_OmicronBA_._2_ is conducive to its immune escape [35].

In State d, the residues that contribute the most to the binding free energy of RBD_OmicronBA_._2_ to ACE2 are R493, H505, Y501, T500, F486, and F456 (Figure 4C). The Q493R, N501Y, and Y505H mutations (−5.6, −4.9, and −4.9 kcal/mol, respectively) contribute strongly to the binding free energy. The reinforced binding of RBD_OmicronBA_._2_ to ACE2 can be confirmed by the R493^RBD^-E35^ACE2^ salt bridge and the H505^RBD^-E37^ACE2^ hydrogen bond (Figure 3N,O).

The S477N, T478K, and E484A mutations are located in region I, the Q493R mutation is located in region II, and the Q498R, N501Y, and Y505H mutations are located in region III. These mutations affect the binding of RBD_OmicronBA_._2_ to ACE2 through the three contact regions (regions I, II, and III, Figure 1E).

**Figure 6:**
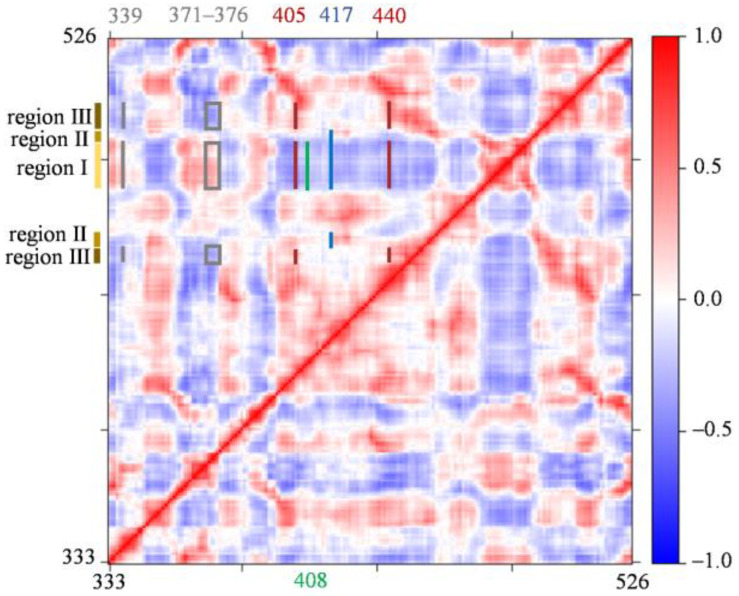
Dynamic cross-correlation map of RBD in the RBD_OmicronBA_._2_-ACE2 system. The color scale is shown on the right changing from red (highly positive correlations) to blue (highly negative correlations).

To analyze the effect of the other mutations (G339D, S371F, S373P, S375F, T376A, D405N, R408S, K417N, and N440K) not located in the contact regions, we performed dynamic cross-correlation matrix (DCCM) analysis for RBD. The DCCM map for RBD_OmicronBA_._2_ is shown in Figure 6. The correlation coefficients of the different residues in RBD are colored from red (1, highly positive correlations) to blue (−1, highly negative correlations). The red color means positive correlations between residues, and the blue color means negative correlations between residues. It is seen that in RBD_OmicronBA_._2_, the G339D, S371F, S373P, S375F, and T376A mutations have positive dynamic correlations with regions I and negative dynamic correlations with region III (see the gray lines and the gray boxes in Figure 6). The D405N and N440K mutations show positive dynamic correlations with region III and negative dynamic correlations with region I (see the dark red lines in Figure 6), the R408S mutation has negative dynamic correlations with region I (see the green line in Figure 6), and the K417N mutation shows positive dynamic correlations with region II and negative dynamic correlations with region I (see the dark blue lines in Figure 6). Thus, the mutations that are not located in the contact regions can affect the RBD_OmicronBA_._2_ recognition and binding to ACE2 through dynamic correlations with regions I, II, and III.

To sum up, the E484A mutation affects the first step in the RBD_Delta_ recognition of ACE2; the Q493R, N501Y, and Y505H mutations in RBD_OmicronBA_._2_ affect the binding free energies between RBD_OmicronBA_._2_ and ACE2 in the last step of recognition; the S477N, T478K, E484A, Q493R, Q498R, N501Y, and Y505H mutations in the contact regions affect the recognition directly; and the G339D, S371F, S373P, S375F, T376A, D405N, R408S, K417N, and N440K mutations not located in the contact regions indirectly affect the RBD_OmicronBA_._2_ recognition of ACE2 through dynamic correlations with the contact regions.

## 3. Materials and Methods

### 3.1. General

The PMEMD module in Amber 20 [36] software was used for all MD simulations. The AMBER FF14SB force field [37] was used for proteins. A nonbonded cutoff distance of 12 Å was used. The Particle Mesh Ewald (PME) algorithm [38] was used to deal with long-range electrostatic interactions, and the SHAKE algorithm [39] was used to constrain bond lengths involving hydrogen atoms. During the simulations, the time step was set to 2 fs. The trajectories were analyzed with CPPTRAJ tools in Amber 20 [36] and VMD [40].

### 3.2. System Setup

In order to simulate the binding process of the spike proteins of different variants of SARS-CoV-2 (WT, Delta and OmicronBA.2) to ACE2, three systems of SARS-CoV-2 spike RBD and human ACE2 were built, which were recorded as the RBD_WT_-ACE2, RBD_Delta_-ACE2, and RBD_OmicronBA_._2_-ACE2 systems. The crystal and cryo-EM structures of the three RBD-ACE2 complexes (PDB ID: 6M0J, 7W9I, 7ZF7) [18,22] were downloaded from the protein data bank. The unnecessary atoms in the crystal and cryo-EM structures were removed, leaving only the RBD and ACE2. The protonation state for titratable residues were determined using the H++ program [41] and the Tleap module of AMBER 20 [36]. The RBD was placed > 40 Å away from ACE2. Then, the system was solvated in a TIP3P water box and neutralized. The dimensions of the RBD_WT_-ACE2, RBD_Delta_-ACE2, and RBD_OmicronBA_._2_-ACE2 systems were approximately 125 Å × 145 Å × 152 Å, 137 Å × 149 Å × 158 Å, and 100 Å × 130 Å × 166 Å.

### 3.3. System Equilibration

Firstly, each system was minimized for 5000 steps with the steepest descent method and then 5000 steps with the conjugate gradient method. Secondly, each system was heated from 0 K to 310 K in 500 ps using the Langevin thermostat [42], and the proteins were constrained with a force constant of 20 kcal·mol^−1^·Å^−2^. Thirdly, three 10 ns equilibrium simulations were performed, and 50 kcal·mol^−1^·Å^−2^, 20 kcal·mol^−1^·Å^−2^, and 5 kcal·mol^−1^·Å^−2^ constraints were added to the proteins. Finally, the whole system was released and equilibrated for 10 ns with no constraints.

### 3.4. Su-GaMD Simulation

The Su-GaMD method [33], which was recently developed by us, was used to simulate the RBD recognition process to ACE2 in the present work. The Su-GaMD approach was a GaMD [34] simulation in which a parameter (*Q*) was supervised by a tabu-like algorithm to accelerate the simulation of the recognition process of RBD to ACE2. The Su-GaMD workflow is depicted in Figure S5. At regular intervals (Δ*t*, 600 ps in the present study), short unbiased GaMD simulation is performed, and the *Q* values of points (a, b, c, d, e, f, g, h) are collected and fitted into a linear function, *f*(*x*) = *m*x. If the slope (*m*) is negative, the parameter *Q* is likely to decrease, and the next short GaMD simulation step starts from the last set of coordinates and velocities produced by the current short GaMD simulation. Otherwise, the current short GaMD simulation is restarted. Short GaMD simulations are perpetuated under supervision until the parameter *Q* is less than the target value *Q*_0_. Only the steps from which the slope (*m*) is negative are saved for analysis. Before the Su-GaMD production run, a series of preparation steps were performed, including a 10 ns short MD simulation to calculate the GaMD acceleration parameters and a 50 ns GaMD equilibration after adding the boost potential. The 50 ns GaMD equilibration was repeated three times to produce three different positions and orientations of RBD to ACE2, i.e., three different starting points for the following Su-GaMD production run. In the Su-GaMD production run, the final coordinates after the 50 ns GaMD equilibration were set as the starting coordinates, and the crystal and cryo-EM structures of the RBD-ACE2 complex (PDBID: 6M0J, 7W9I, and 7ZF7) were set as the targeting coordinates. During the Su-GaMD simulations, the RMSDs calculated on the heavy atoms in the main chain of the receptor-binding motif (RBM, residues 438–506 in RBD, as shown in Figure 1A) relative to the targeting structures (RMSD_RBM_, i.e., the *Q*) were supervised until the RMSD_RBM_ were less than 1 Å (i.e., the target value *Q*_0_).

### 3.5. Binding Free Energy Calculations

The binding free energies of RBD to ACE2 were calculated using the molecular mechanics generalized born surface area (MM/GBSA) [43] approach. All of the parameters were set to the default values in the calculations.

### 3.6. Dynamic Cross-Correlation Matrix (DCCM) Analysis

Bio3D [44], an R package, was used to generate the DCCM map to explore the interrelationships and effects between the amino acid residues in RBD. Cross-correlations of residues in RBD were calculated based on mutual information between all Cα atoms. In the DCCM map, a positive value indicates that the two Cα atoms have the same direction of motion, while a negative value indicates that the two Cα atoms have opposite directions of motion. The values calculated by DCCM ranged from 1.0 for a complete positive correlation to −1.0 for a complete negative correlation.

## 4. Conclusions

In the present work, the recognition and binding process of SARS-CoV-2 spike RBD of the WT, Delta, and OmicronBA.2 variants to human ACE2 were simulated using the Su-GaMD approach, which was developed recently by us. The possible recognition pathways and important intermediate states of the RBD-ACE2 recognition process were identified, the RBD_WT_-ACE2, RBD_Delta_-ACE2, and RBD_OmicronBA_._2_-ACE2 complexes were reconstructed, and the effects of the mutations in RBD_Delta_ and RBD_OmicronBA_._2_ to RBD-ACE2 recognition and binding were discussed.

In the RBD_WT_-ACE2 recognition process, RBD_WT_ is anchored to ACE2 through region I first, and then region III at the other end of RBD_WT_ is locked to ACE2 as well. Finally, region II in the middle of the RBD_WT_ is attached to ACE2 and reinforces the RBD_WT_-ACE2 binding. This recognition process revealed by our Su-GaMD simulation aligns well with the anchor–locker mechanism. In the RBD_Delta_-ACE2 system and the RBD_OmicronBA_._2_-ACE2 system, RBD_Delta_ and RBD_OmicronBA_._2_ recognize ACE2 in the chronological order of region I, II, and III, which is slightly different from RBD_WT_.

The trend in the calculated binding free energies in State d of RBD_WT_-ACE2, RBD_Delta_-ACE2, and RBD_OmicronBA_._2_-ACE2 correlates well with the increased infectivity of OmicronBA.2 in comparison with other SARS-CoV-2 variants. By analyzing the key intermediate states in the binding process between RBD_Delta_ and ACE2, it is found that the T478K mutation affects the first step in the RBD_Delta_ recognition of ACE2, while the L452R mutation affects, not directly but through its nearby Y449, the binding between RBD_Delta_ and ACE2 in the last step of recognition. For the RBD_OmicronBA_._2_-ACE2 system, the E484A mutation affects the first step in the RBD_OmicronBA_._2_ recognition of ACE2; the Q493R, N501Y, and Y505H mutations in RBD_OmicronBA_._2_ affect the binding free energy between RBD_OmicronBA_._2_ and ACE2 in the last step of recognition; the S477N, T478K, E484A, Q493R, Q498R, N501Y, and Y505H mutations in the contact regions affect the recognition directly; and the G339D, S371F, S373P, S375F, T376A, D405N, R408S, K417N, and N440K mutations that are not located in the contact regions indirectly affect the RBD_OmicronBA_._2_ recognition of ACE2 through dynamic correlations with the contact regions.

Overall, the current computational study provides important theoretical insights into the molecular mechanisms involved in the way the SARS-CoV-2 spike RBD recognizes human ACE2. The results provide a detailed explanation of the impact of mutations in spike RBD for virus recognition and infectivity in humans. We hope this study provides valuable information and shed light onto the development of new drugs to inhibit SARS-CoV-2 entry into the human body.

## Data Availability

The data presented in the study are included in the article and Supplementary Materials. Further inquiries can be directed to the corresponding authors.

## Supplementary Materials

The following supporting information can be downloaded at: https://www.mdpi.com/article/10.3390/molecules29081875/s1. Figure S1: The initial state, State a, and the time-dependent RMSD_RBM_ of the Su-GaMD simulations from three different starting points for the recognition process of RBD_WT_ to ACE2; Figure S2: The starting point (which is the same as in Figure 2A) and the time-dependent RMSD_RBM_ of the 1000 ns cMD simulation for the ACE2 recognition process of RBD_WT_; Figure S3: The initial state, State a, and the time-dependent RMSD_RBM_ of the Su-GaMD simulations from three different starting points for the ACE2 recognition process of RBD_Delta_; Figure S4: The initial state, State a, and the time-dependent RMSD_RBM_ of the Su-GaMD simulations from three different starting points for the ACE2 recognition process of RBD_OmicronBA_._2_; Figure S5: Workflow of the Su-GaMD simulation; Video S1: The RBD_WT_-ACE2 recognition process observed in the Su-GaMD simulation; Video S2: The RBD_Delta_-ACE2 recognition process observed in the Su-GaMD simulation; Video S3: The RBD_OmicronBA_._2_-ACE2 recognition process observed in the Su-GaMD simulation.
