# Supplementary material for: The Recognition Pathway of the SARS-CoV-2 Spike Receptor-Binding Domain to Human Angiotensin-Converting Enzyme 2
Source: Molecules. 2024 Apr 19;29(8):1875. doi: 10.3390/molecules29081875 (PMC11054751; doi:10.3390/molecules29081875)
Supplement: Supplementary file 1 [file molecules-29-01875-s001.zip › Molecules-SI-rev.pdf]

Supplementary Materials

The Recognition Pathway of the SARS-CoV-2 Spike Receptor-Binding Domain to Human Angiotensin-Converting Enzyme 2

Can Peng [1], Xinyue Lv [1], Zhiqiang Zhang [2], Jianping Lin [1,*] and Dongmei Li [1,*]

[1] State Key Laboratory of Medicinal Chemical Biology, College of Pharmacy and Tianjin Key Laboratory
of Molecular Drug Research, Nankai University, Haihe Education Park, 38 Tongyan Road, Tianjin 300350, China; 2120211306@mail.nankai.edu.cn (C.P.); 2120211384@mail.nankai.edu.cn (X.L.)
[2] Xiongan Institute of Innovation, Xiong'an New Area 070001, China; zhangzhiqiang@xii.ac.cn
* Correspondence: jianpinglin@nankai.edu.cn (J.L.); dongmeili@nankai.edu.cn (D.L.)

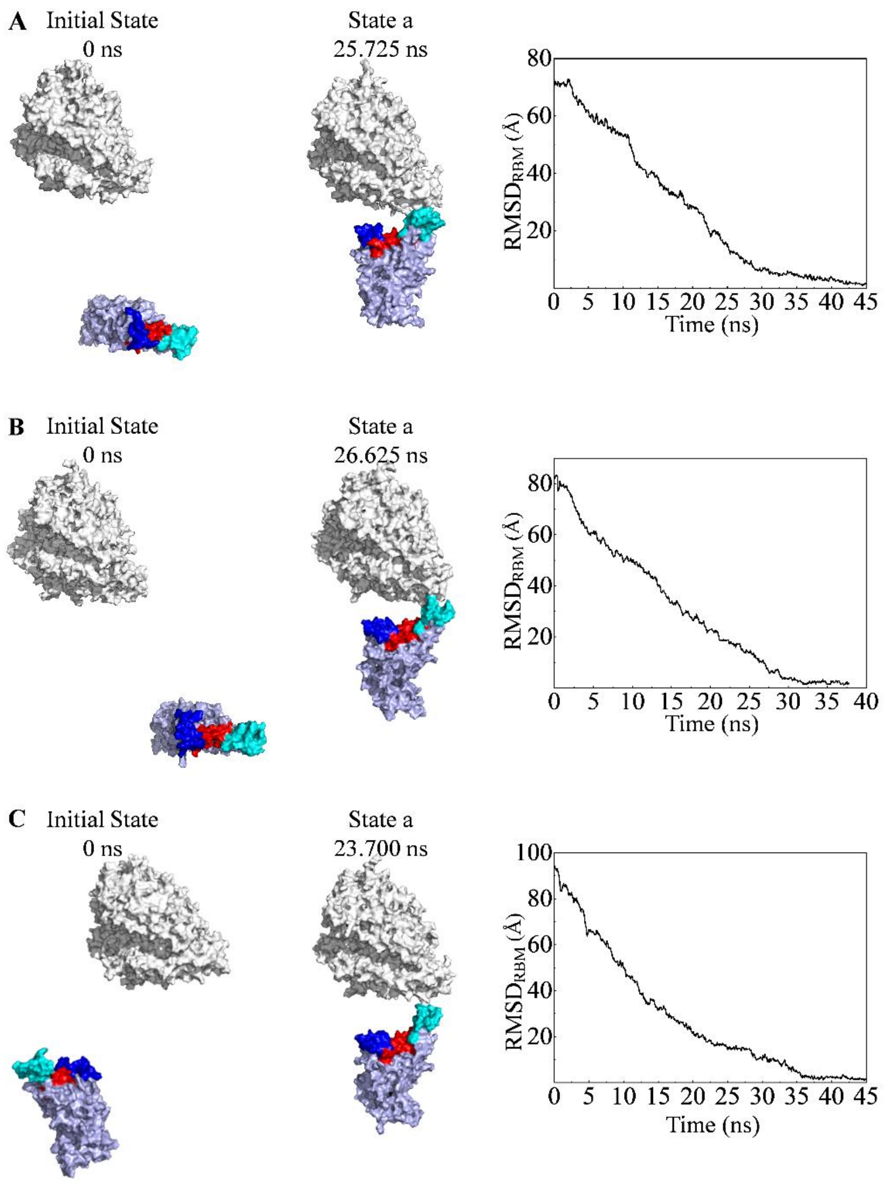

Figure S1. The initial state, State a and the time-dependent $RMSD_{RBM}$ of the Su-GaMD simulations from three different starting points for the recognition process of RBD_{WT} to ACE2.

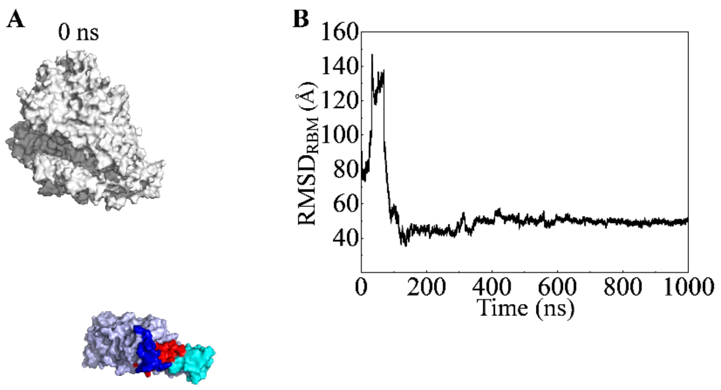

Figure S2. (A) The starting point (which is the same as in Figure 2A) and (B) the time-dependent $RMSD_{RBM}$ of the 1000-ns cMD simulation for the recognition process of RBD_{WT} to ACE2.

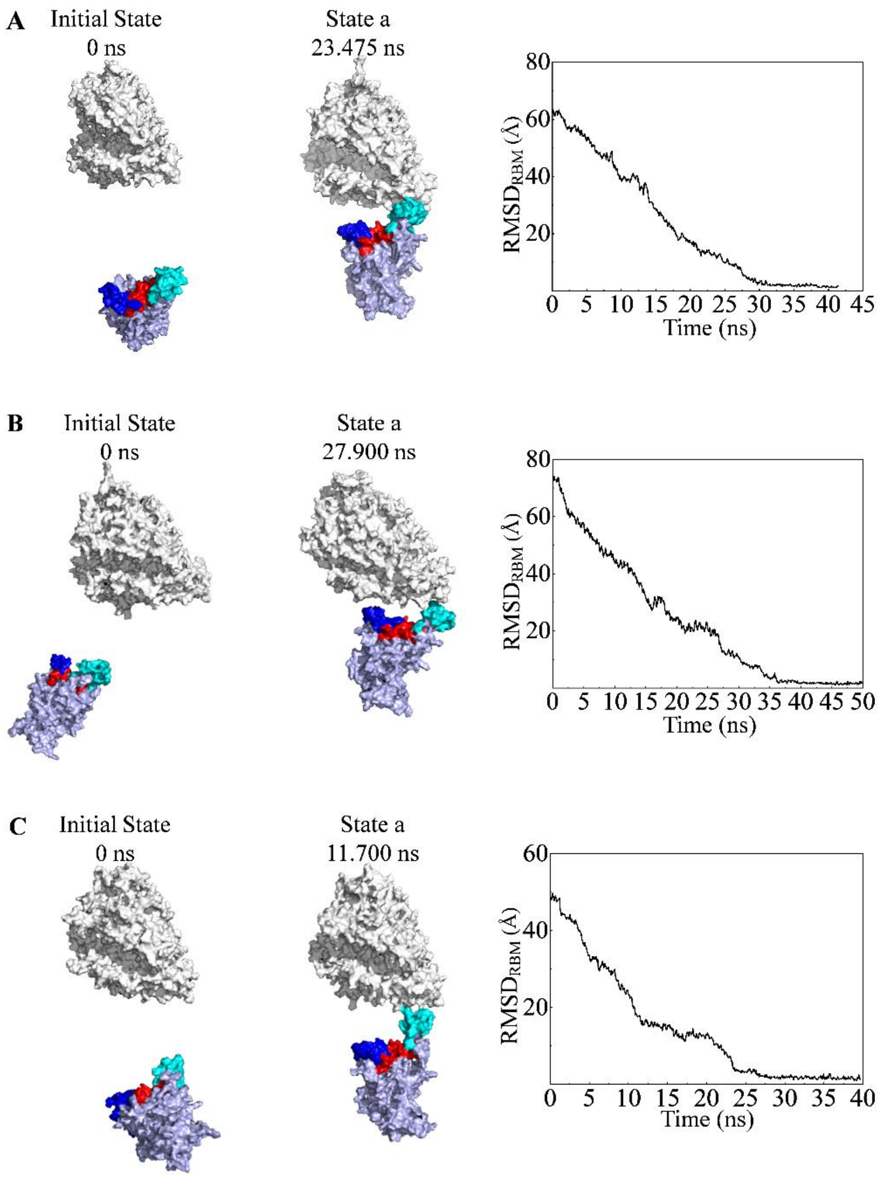

Figure S3. The initial state, State a and the time-dependent $RMSD_{RBM}$ of the Su-GaMD simulations from three different starting points for the recognition process of RBD_{Delta} to ACE2.

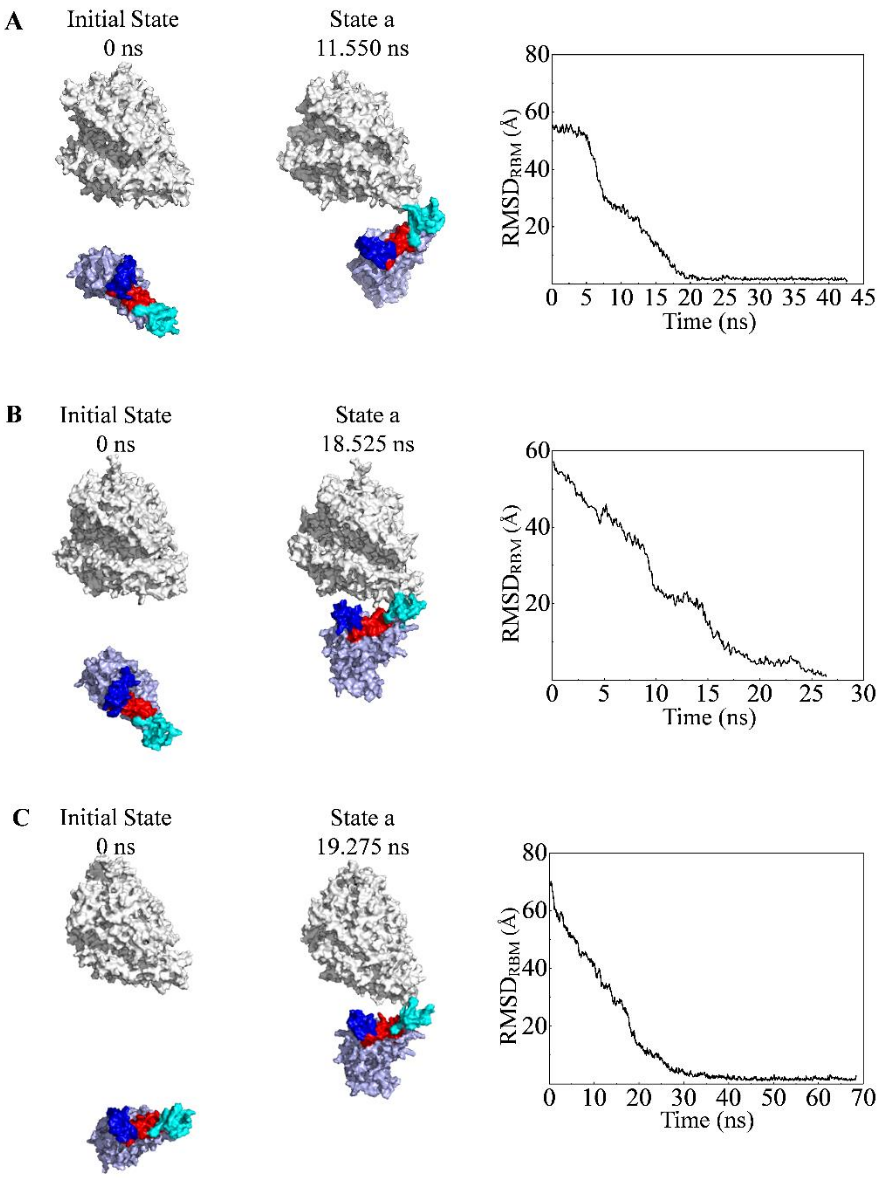

Figure S4. The initial state, State a and the time-dependent $RMSD_{RBM}$ of the Su-GaMD simulations from three different starting points for the recognition process of $RBD_{OmicronBA.2}$ to ACE2.

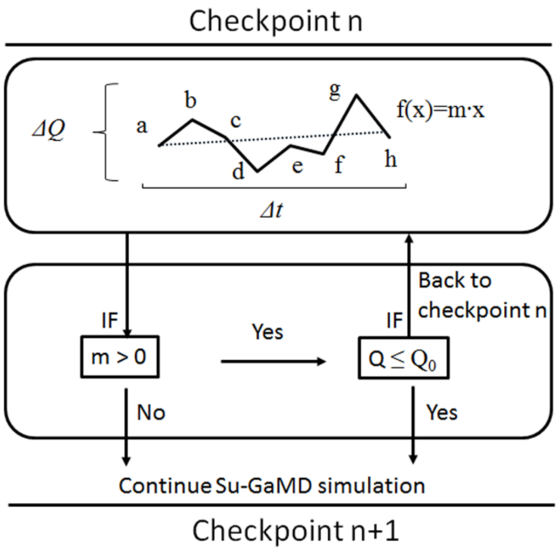

Figure S5. Workflow of the Su-GaMD simulation.
